# From maturation to degeneration: Distinct biological trajectories in bicuspid and tricuspid aortopathy

**DOI:** 10.1113/EP092864

**Published:** 2026-10-13

**Authors:** Riaz Akhtar, Daniel Olaf Riley, Jillian Madine, Mark Field

**Affiliations:** ^1^ Department of Materials Design and Manufacturing Engineering School of Engineering University of Liverpool Liverpool UK; ^2^ Department of Biochemistry, Cell and Systems Biology Institute of Systems, Molecular and Integrative Biology University of Liverpool Liverpool UK; ^3^ Department of Cardiac Surgery Liverpool Heart and Chest Hospital Liverpool UK

**Keywords:** aortopathy, ascending thoracic aortic aneurysms, bicuspid aortic valve, medial degeneration, tricuspid aortic valve, vascular ageing

## Abstract

Ascending thoracic aortic aneurysm formation differs fundamentally between patients with bicuspid (BAV) and tricuspid (TAV) aortic valve phenotypes, yet current clinical management largely relies on shared diameter thresholds, although clinical and scientific data fail to correlate with diameter and risk of rupture. Over the past two decades, histological, biochemical and biomechanical studies have revealed unique differences between dilated tissue properties from patients with BAV‐ and TAV‐associated idiopathic degenerative ascending aortic aneurysms even at similar aortic diameters. This review focuses on histological, biochemical and biomechanical differences between BAV and TAV aortopathy with a focus on ageing. Idiopathic degenerative TAV aortopathy is characterised by medial degeneration with elastic lamellar fragmentation and loss, smooth muscle cell depletion, medial fibrosis, mucoid extracellular matrix accumulation and inflammatory features. These degenerative changes correlate with ageing and, in region‐specific mechanical testing, with reduced delamination resistance and age‐dependent biomechanical weakening. In contrast, BAV aortopathy frequently demonstrates relative preservation of medial architecture despite similar dilatation, alongside altered collagen maturation, and distinct osteogenic/microcalcification signatures. Lifespan analyses indicate that TAV follows a maturation–stabilisation–degeneration trajectory, whereas BAV exhibits aberrant vascular maturation without a true stabilisation phase. Importantly, aortic diameter correlates poorly with histological severity or biomechanical vulnerability, particularly in BAV. Collectively, these data support the concept that ascending thoracic aortic aneurysms in BAV and idiopathic degenerative TAV patients represent biologically divergent aortopathies requiring phenotype‐informed risk stratification beyond diameter alone.

## Abstract (highlights)

- **What is the topic of this review?**
  Structural, biomechanical and biochemical differences in ascending thoracic aortic aneurysm tissue in patients with bicuspid aortic valve (BAV) as compared to those with tricuspid aortic valves (TAV) and non‐syndromic disease.
- **What advances does it highlight?**
  The review highlights that BAV aortopathy is often associated with a preserved aortic wall in terms of key extracellular matrix components In contrast, TAV‐associated aneurysms have been reported to exhibit more pronounced extracellular matrix degeneration, including greater disruption of elastic fibres. Surgical risk stratification for ascending thoracic aortic aneurysms should be based on disease subtypes and not on aortic diameter alone.

## 1 INTRODUCTION

Ascending thoracic aortic aneurysms (ATAAs) are often asymptomatic and can be fatal if rupture or dissection occurs. Surgical repair of ascending aortic aneurysms has largely been guided by a single measure, the aortic diameter. This means that elective surgical repair is offered at an aortic diameter of ≥5.5 cm in asymptomatic patients without additional risk factors (Isselbacher et al., 2023). In older patients, ATAAs often occur due to age‐related changes where cystic medial degeneration weakens the aortic wall (Salameh et al., 2018), and prevalence is associated with smoking and high blood pressure. In younger patients, ATAA is typically associated with genetic causes including connective tissue disorders. For example, Marfan syndrome, and Loeys–Dietz syndrome (Evangelista, 2010). However, the majority of ATAAs are not related to any specific aetiology and can be defined as non‐syndromic or idiopathic degenerative aneurysms (Kirsch et al., 2006). Additionally, ATAAs can be associated with bicuspid aortic valve (BAV), the most common congenital heart defect worldwide. BAV affects 1–2% of the population and is 3 times more common in males than females (Mordi & Tzemos, 2012). The aortic valve in BAV patients only has two cusps (bicuspid) instead of the normal three (tricuspid). The valve morphology in BAV can be classified by cusp symmetry, sinus configuration and commissural orientation (Michelena, 2022), and four clusters of BAV aortopathy have been identified (Fazel et al., 2008; Stock et al., 2019) (Figure 1).

**FIGURE 1:**
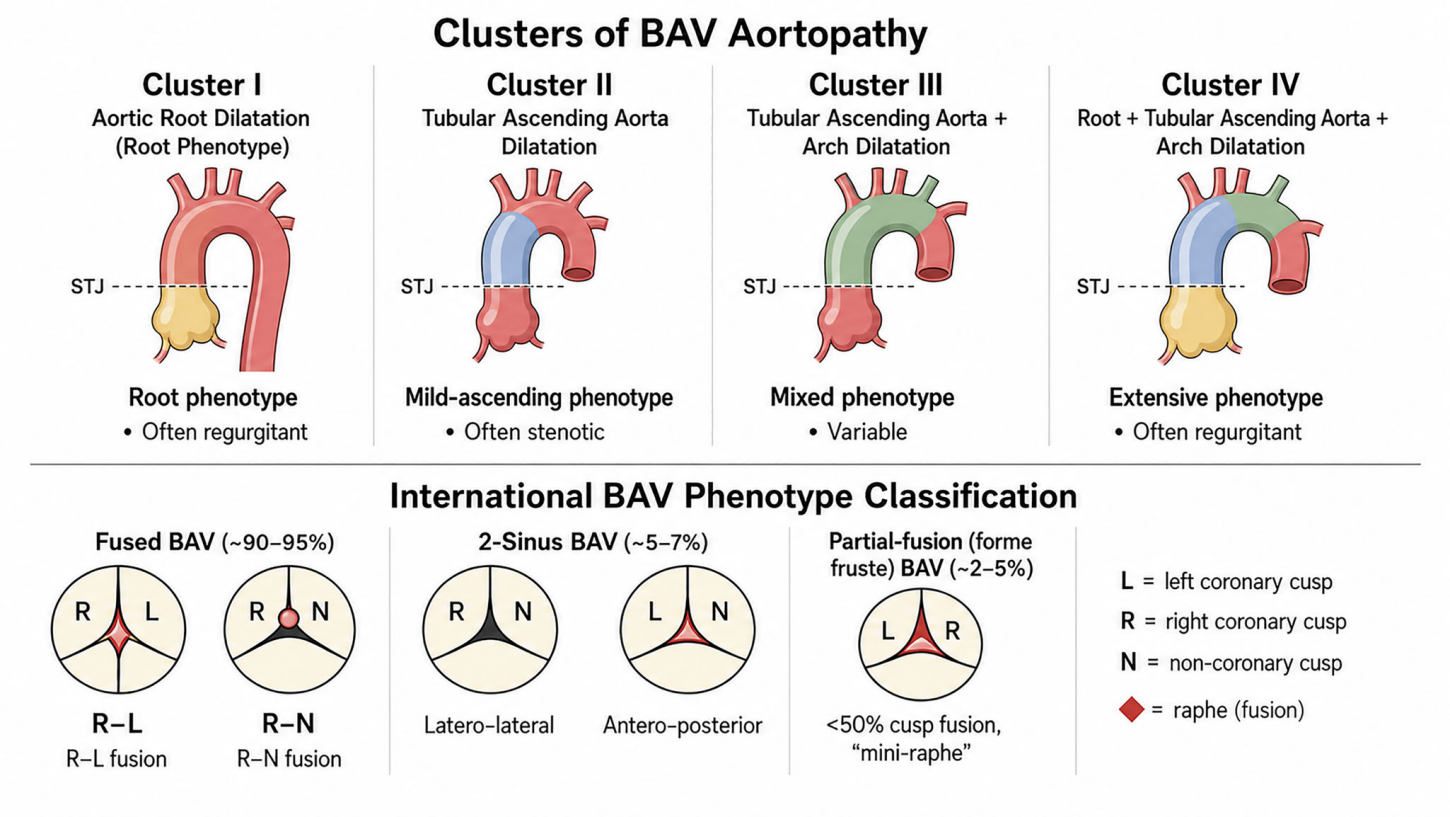
International consensus BAV morphology and associated aortopathy clusters. The top row illustrates the four principal patterns of BAV‐A: Cluster I, isolated dilatation of the aortic root at the sinuses of Valsalva, below the sinotubular junction (STJ); Cluster II, isolated dilatation of the tubular ascending aorta above the STJ; Cluster III, dilatation of the tubular ascending aorta and aortic arch; and Cluster IV, extensive dilatation involving the aortic root, tubular ascending aorta and arch (Fazel et al., 2008). Coloured regions indicate the affected aortic segments. The second row depicts bicuspid aortic valve morphology according to the international consensus classification: fused BAV (∼90–95%), characterised by fusion of two cusps within three sinuses (typically right–left or right–non fusion); 2‐sinus BAV (∼5–7%), comprising two symmetric sinuses with 180° commissural orientation (latero‐lateral or antero‐posterior configuration); and partial‐fusion (forme fruste) BAV (∼2–5%), defined by < 50% cusp fusion with a ‘mini‐raphe’ (Michelena, 2022; Michelena et al., 2021). The international consensus classification standardises imaging and clinical reporting but the morphology‐based Sievers surgical classification defined by number of raphes (Type 0, 1, 2) and cusp fusion patterns (e.g., Type 1 LR, RN, LN) is most widely reported in surgical and pathological studies (Sievers & Schmidtke, 2007). The international consensus defines three aortopathy phenotypes (Michelena et al., 2021), ascending phenotype, root phenotype and extended phenotype, as compared to the four clusters defined by Fazel and shown in this figure. In practice, the root phenotype is equivalent to Fazel Cluster I, ascending phenotype to Fazel cluster II and the extended phenotype encompasses aspects of Fazel Clusters III and IV.

ATAAs in BAV patients as compared to idiopathic degenerative aneurysms (non‐syndromic) in tricuspid aortic valve (TAV) patients provide a unique human model to determine how congenital versus acquired disturbances in haemodynamics and vascular ageing lead to distinct biomechanical and histopathological trajectories within the thoracic aorta. TAV is the normal anatomical configuration in approximately 99% of the population and idiopathic aneurysms in this group represent an acquired degenerative disease developing from a previously normal aortic wall. Whilst clinical guidelines (Isselbacher et al., 2023; Praz et al., 2025) typically recommend surgical repair of aneurysms in TAV at an ascending aortic diameter of ≥5.5 cm or 5 cm with certain risk factors, historically BAV‐associated aneurysms were recommended for repair at smaller diameters due to potential risk of dissection or rupture (Borger et al., 2018). However, evidence suggests that the threshold for surgical repair of ATAAs in BAV should not differ from that of TAV (Zafar et al., 2024). We can argue that BAV and TAV aneurysms are not just size‐based surgical entities, but distinct vascular phenotypes associated with different biomechanical and haemodynamic drivers. Given the limitations of diameter‐based risk stratification (Acharya et al., 2021), there is a need for multi‐scale integration of various features of the cardiovascular system including the valve, blood flow, aortic wall stress and tissue remodelling to better understand and predict the risk of dissection or rupture in ATAAs associated with BAV and TAV.

Over the past two decades, there have been numerous studies which have examined the pathobiology of BAV‐associated aortopathy (BAV‐A) and postulated that differing mechanisms drive aortic dilatation as compared to idiopathic degenerative ATAAs in TAV patients (TAV‐associated aortopathy, TAV‐A) (Acharya et al., 2021). It is clear that a multi‐faceted approach is needed to fully understand the role of BAV‐A with consideration of valve morphology, wall stress and flow‐induced tissue remodelling (Forte & Della Corte, 2014) (Figure 2).

**FIGURE 2:**
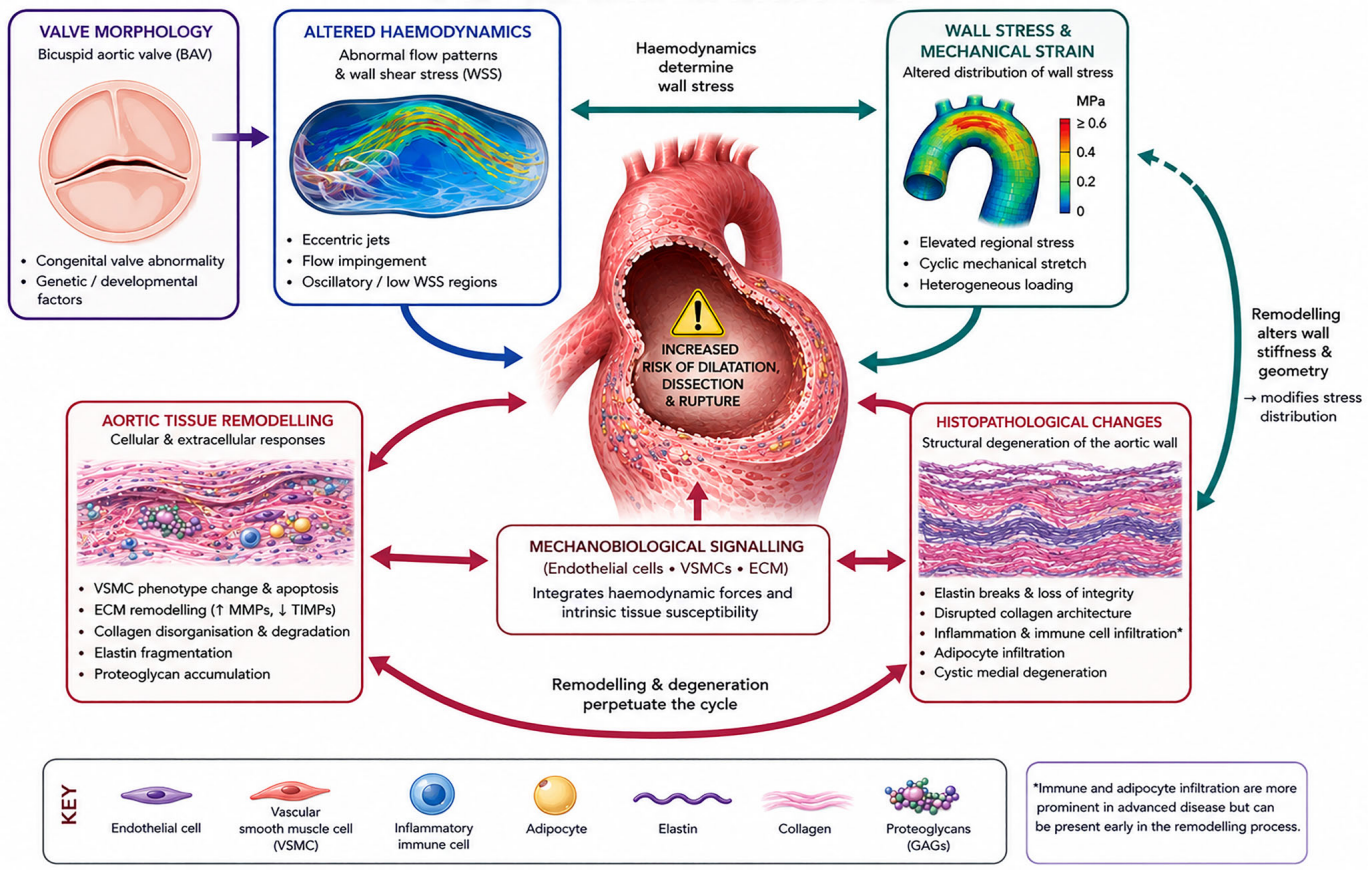
Rupture risk in BAV‐A is driven by multiple factors including aortic tissue remodelling, inflammation, blood flow and aortic wall stress.

As highlighted in Figure 2, abnormal valve morphology alters systolic flow patterns and regional wall shear stress. These haemodynamic forces interact with intrinsic abnormalities of the aortic wall to regulate collagen remodelling, elastin degradation, inflammatory cell recruitment, and smooth muscle cell phenotype. Consequently, the regional histopathological changes observed in BAV‐associated aortopathy likely reflect the combined effects of altered biomechanics acting upon a developmentally susceptible aortic wall.

There are numerous comprehensive reviews which focus on the anatomy, natural history, pathophysiology, genetics and clinical aspects of BAV disease (e.g., Mordi & Tzemos, 2012; Salzillo et al., 2025). This review seeks to address a gap in understanding the tissue‐level alterations in the ascending aorta in BAV and how our understanding has evolved over the past two decades. We focus on BAV‐A relative to idiopathic degenerative TAV‐A. As well as the natural ageing process, TAV‐A are typically related to lifestyle factors such as smoking, and hence they serve as a useful comparator to the congenital BAV condition as the underlying disease mechanisms and clinical profiles of aneurysm development differ between these two groups. Clinically, there is now more agreement that the risk associated with BAV‐A is more heterogeneous than historically perceived rather than uniformly aggressive in all patients. (Borger et al., 2018). Through examining the histopathology, biomechanics and biochemistry of BAV‐A compared to TAV‐A, this review will address this question from a scientific perspective, in terms of our understanding of the tissue composition and microstructure. The key questions we seek to address with this review are: (1) Are there histopathological, biochemical and biomechanical differences between BAV‐A and idiopathic degenerative TAV‐A? (2) How do these relate to aortic diameter and the natural ageing process?

A 2012 review by Losenno et al. synthesised evidence and reported that BAV‐A is linked to early smooth muscle cell loss and extracellular matrix (ECM) disruption, hence weakening the aortic wall and predisposing the aorta to progressive dilatation and aneurysm (Losenno et al., 2012). However, by examining a wider body of literature, especially more recent papers, it is clear that idiopathic degenerative TAV‐A manifests like a classic age‐related degenerative medial disease whilst BAV‐A often dilates with relatively preserved histological and mechanical features but with signs of distinct biological remodelling. This is highlighted in Table 1, which provides a summary of key studies that have investigated the histological and/or biomechanical properties of aortic tissue in BAV‐A and TAV‐A patients.

**TABLE 1:** A summary of key studies which have examined the histopathology, biochemical and/or biomechanical properties of BAV‐A and TAV‐A tissues.

Reference	Phenotype	Sample size (population size and cohort split), sex, age and discrepancies (if noted)	Histological/biochemical finding	Biomechanical findings
Alloisio et al. (2025)	BAV‐A and TAV‐A	*n* = 36 (BAV‐A, *n* = 16; TAV‐A, *n* = 20), 29 males, 7 females Mean age: BAV‐A, 69.6; TAV‐A, 56.3	Collagen density correlated positively with peak load, indicating that collagen content contributes significantly to aneurysmal vessel wall strength.	BAV‐A tissue exhibited greater mechanical resistance than TAV tissue. Greater fracture energy was needed to full propagate through BAV‐A than TAV‐A.
Bauer et al. (2002)	BAV‐A and TAV‐A	*n* = 168 (BAV‐A, *n* = 107; TAV‐A, *n* = 61), 76 males, 31 females. Mean age: 60.9.	Elastic lamellae were thinner and had wider spacing in BAV‐A, with spacing increasing with diameter	N/A
Brecs et al. (2024)	BAV‐A and TAV‐A	*n* = 26 (BAV‐A, *n* = 12; TAV‐A, *n* = 14; Controls; *n* = 13), 18 males and 8 females from aneurysm groups. Control sex data not provided. Median age: BAV, 59.5; TAV, 68.25.	BAV‐A tissues exhibited significant collagen fragmentation vs. TAV‐A and controls, suggesting greater structural deterioration of the extracellular matrix. TAV‐A showed increased overall collagen density with thickening between media and adventitia layers, and disorganised collagen fibres. BAV‐A specimens showed elevated adipocyte deposits and greater immune cell infiltration compared with TAV‐A.	BAV‐A showed higher biomechanical stress and strain compared with controls and TAV‐A.
Chim et al. (2020)	BAV‐A and TAV‐A	*n* = 39 (BAV‐A, *n* = 15; TAV‐A, *n* = 15, Controls; *n* = 9), 15 males and 15 females for the aneurysm groups, 7 males and 2 females for control groups. Mean age: BAV‐A, 61.0; TAV‐A, 66.4.	BAV‐A tissues had more preserved but compact elastic lamellae whereas TAV‐A had sparse elastic lamellae (assessed via histology).	BAV‐A tissues showed ∼20% higher elastic modulus as determined by nanoindentation.
Collins et al. (2008)	BAV‐A and TAV‐A	*n* = 111 (BAV‐A, *n* = 34; TAV‐A, *n* = 71; Marfan; *n* = 12), 74 males, 37 females. Mean age: BAV‐A, 56.7; TAV‐A, 59.9.	Semi‐quantitative analysis revealed greater elastin fragmentation and loss in TAV‐A samples compared to BAV‐A. Overexpression of MMP‐2 in BAV patients reported. TAV‐A exhibited more severe medial degeneration, including greater elastic fibre fragmentation and loss, whereas BAV‐A exhibited relative preservation of elastic tissue despite abnormal elastic lamellar organisation.	N/A
Phillippi et al. (2014)	BAV‐A and TAV‐A	*n* = 103 (TAV, 23; TAV‐A, 26; BAV, 15; BAV‐A, 39), 85 males, 18 females. Median age: TAV, 54; TAV‐A, 58.5; BAV, 56; BAV‐A, 54. Note, histology and biochemistry reported on a subset of the patient cohort. Details are in the figure captions of the paper.	BAV‐A showed aligned collagen and elastin fibres resembling normal aorta, whereas TAV‐A had a disorganised matrix. MMP‐2/9 activity was higher in TAV‐A, while SMC density was greater in BAV‐A.	N/A
Wågsäter et al. (2013)	BAV‐A and TAV‐A	*n* = 109 (biochemical/histological analysis subset, *n* = 56; BAV‐A, *n* = 12; BAV, *n* = 16; TAV‐A, 13; TAV, 15), 80 males and 29 females. Mean age: 61 (BAV/TAV mean age is provided by aortic size in the paper).	Overall collagen content (total amount) was similar between BAV and TAV non‐dilated aortas. TAV aneurysms showed increased collagen turnover, evidenced by lower collagen half‐life and more extensive fibrotic change. BAV aneurysms did not have the expected increase in collagen turnover with dilatation. Collagen turnover rates in BAV nondilated vs. dilated aortas were similar, indicating a different disease mechanism.	N/A
Pisano et al. (2020)	BAV‐A and TAV‐A	*n* = 26 (BAV‐A, *n* = 14; TAV‐A, *n* = 12; Controls, *n* = 6), 17 males, 9 females. Mean age: BAV‐A, 62; TAV‐A, 76.5.	GAG levels were similar in BAV‐A and TAV‐A but lower in controls Both BAV‐A and TAV‐A had loss and fragmentation of elastic fibres compared with non‐aneurysmal controls. But, there was no significant difference in elastic fibre characteristics between BAV and TAV. Collagen density was higher in TAV, while MMP‐2 expression was elevated in BAV‐A.	BAV‐A tissue showed higher peak wall stress and lower Young's modulus than TAV‐A, indicating greater deformability.
Nightingale et al. (2022)	Ascending aortic aneurysms	*n* = 41 (BAV‐A, *n* = 24; TAV‐A, *n* = 17), 27 males, 14 females. Mean age: BAV‐A, 57.12; TAV‐A, 67.88.	Reduced elastin and proteoglycan (PG) content and increased elastin fragmentation.	Low strain tangential modulus (LTM) and transition zone onset (TZo) stress were correlated with reduced strength as well elastin content.
Neave et al. (2022)	BAV‐A	*n* = 15, 14 males, 1 female Mean age: 57.27.	COL1A1 and COL3A1 expression related to the collagen‐I content of the tissue but did not independently correlate with mechanical performance. Collagen type rather than collagen quantity was most important for mechanical behaviour.	Collagen‐I content in the aortic wall correlated with improved mechanical properties Older BAV‐A tissues exhibited greater stiffness and lower ultimate strength.
García‐Herrera et al. (2012)	BAV‐A and TAV‐A	*n* = 43 (BAV‐A, *n* = 11; TAV‐A, *n* = 11; Controls, *n* = 21), sex distribution not fully reported. Mean Age: BAV‐A, 57; TAV‐A, 60; Controls, 41 Demographic data were extracted from Table 1 because these data are internally consistent. Total stated sample: *n* = 49. Participant‐level demographic tables include only *n* = 43.	N/A	No significant difference between BAV and TAV aneurysm tissues in terms of tensile strength and stretch at failure when age‐matched. Age was the dominant determinant of rupture mechanics.
Stern et al. (2019)	BAV‐A and TAV‐A	*n* = 77 (BAV‐A, *n* = 19; TAV‐A, *n* = 28; Controls, *n* = 30) Mean age: TAV‐A, 65.7; BAV‐A, 52.8; Controls, 53.3 The footnote to Table 1 reports *n* = 26, 26 and 15 (Control, TAV‐A and BAV‐A, respectively), but this conflicts with the table itself, the Results section and the figure legends. These smaller numbers appear to refer specifically to the aortic regurgitation data.	TAV‐A shows severe wall degeneration with collagen loss and elastic fibre damage, while BAV‐A shows milder changes but higher osteopontin and age‐related calcification, indicating distinct disease mechanisms.	N/A
Karalko et al. (2021)	BAV‐A and TAV‐A	*n* = 376 (BAV‐A, *n* = 209; TAV‐A, *n* = 167), 268 males, 108 females. Mean age: BAV‐A, 56; TAV‐A, 62	TAV‐A showed medial degeneration with elastic fibre loss, mucoid matrix accumulation, and SMC loss, while BAV‐A often had milder or no abnormalities. No age‐dependent histological progression was detected in BAV. Only SMC loss showed a weak age dependence in TAV. Aortic diameter correlated poorly with histological degeneration, especially in BAV.	N/A
Bechtel et al. (2003)	BAV‐A and TAV‐A	*n* = 65 (BAV‐A, *n* = 26, TAV‐A, *n* = 39), 43 males, 22 females Mean age: BAV‐A, 53; TAV‐A, 57	Histopathological severity in ascending aortic aneurysms correlates with aortic diameter in both BAV and TAV patients, but correlates with age only in TAV, with BAV patients exhibiting significantly lower overall wall degeneration despite similar degrees of dilatation.	N/A
Tucker et al. (2022)	BAV‐A and TAV‐A	The study included 314 TAV patients in the overall surgical cohort, 77 for BAV and 168 for connective tissue disorder. However, the number of specimens included in the histological analyses was not explicitly reported. Age and sex for the histology subset were therefore not extractable.	BAV‐A had less severe medial degeneration and better preservation of elastic lamellar structure and medial architecture. TAV‐A had more advanced medial degeneration with greater elastic fibre fragmentation, SMC loss and medial fibrosis. The connective tissue disorder group had the most severe and diffuse medial degeneration.	N/A
Mennander et al. (2022)	BAV‐A and TAV‐A	*n* = 67 (BAV‐A, *n* = 33; TAV‐A, *n* = 34), 50 males, 17 females Mean age: BAV, 64; TAV, 68	TAV‐A exhibited a more degenerative medial phenotype, while BAV‐A showed relative preservation of wall structure. This included SMC nuclei loss, medial fibrosis and elastic fibre thinning.	N/A
Zhang et al. (2024)	BAV‐A and TAV‐A	*n* = 23 (BAV‐A, *n* = 10; TAV‐A, *n* = 13), hypertensive patients only, 17 males, 6 females. Mean age: BAV‐A, 69; TAV‐A, 72.	TAV‐A had more severe elastic lamellar disruption and fragmentation relative to BAV‐A.	Peeling tests conducted to examine delamination strength. TAV‐A were mechanically more vulnerable to dissection propagation, particularly along the longitudinal direction.
Knochenhauer et al. (2024); not a full paper – congress abstract	BAV‐A and TAV‐A	*n* = 97 (BAV‐A, *n* = 29, TAV‐A, *n* = 68) No age or sex information provided.	TAV‐A patients had a higher rate of calcific atherosclerosis, severe mucoid extracellular matrix accumulation, and significantly higher rates of severe elastic fibre fragmentation relative to BAV‐A. TAV‐A also had more severe elastic fibre thinning and extensive elastic fibre disorganisation.	Aortic distensibility was measured with transthoracic echocardiography and no significant difference was found between BAV and TAV patients.
Fletcher et al. (2022)	BAV‐A and TAV‐A	*n* = 63 (excluding dissections) (BAV‐A, *n* = 28; TAV‐A, *n* = 17; Controls; *n* = 18), 43 males, 20 females Mean age: BAV‐A, 59.9; TAV‐A, 65.7; Controls: 71.6	TAV‐A had more cases of advanced medial degeneration with greater elastin loss. In BAV‐A, microcalcification profile was more associated with mild aortopathy. Microcalcification serves as a protective mechanism for elastin preventing more serious disease seen in BAV‐A.	A positive correlation between tissue elastic modulus and microcalcification was observed.
Grewal et al. (2014)	BAV‐A and TAV‐A	*n* = 59 (TAV, *n* = 11; TAV‐A, *n* = 12; BAV, *n* = 17; BAV‐A, *n* = 19), sex split not provided Mean age: TAV, 64.5; TA‐A, 72.3; BAV, 55.8; BAV‐A, 60.7	In non‐dilated tissue, BAV had thicker media with well‐organised lamellae and significantly reduced expression of contractile VSMC markers. Higher inflammation in dilated TAV.	N/A

All of the studies are full length papers except Knochenhauer et al., 2024, which is a congress abstract. Where authors had access to non‐dilated tissue they are referred to by the valvular phenotype (BAV or TAV). The TAV cohorts compared directly in these studies with BAV patients are idiopathic degenerative aneurysms but it should be noted that genetic sequencing is rarely carried out routinely at most surgical centres and hence any genetic predisposition in any TAV cohort cannot be ruled out.

Although there are some conflicting trends reported in Table 1, the overall trends can be summarised here. The studies suggest that TAV aneurysms have more medial degeneration than BAV. Greater elastin fragmentation and loss is reported in TAV, but BAV may have intrinsically abnormal elastic lamellar architecture (e.g., as determined by morphometric analysis by Bauer et al., 2002). Collagen architecture appears to be more preserved in BAV but with impaired biosynthesis. Age has a significant impact on the biomechanical properties and a range of different mechanical endpoints. For example, fracture energy, elastic modulus, and tensile strength limit direct comparison. However, taken together it can be inferred that TAV tissue is more biomechanically compromised than BAV.

The overwhelming trends that emerge from these studies (Table 1) is that in TAV‐A, a degenerative medial phenotype, is observed with histopathology studies, whereas BAV‐A is characterised by dilatation without degeneration. Multiple studies including our own work (Chim et al., 2020) have found relatively preserved medial components and less severe elastin loss in BAV‐A even at similar diameters. Our microcalcification work (Fletcher et al., 2022) showed that microcalcification acts as a severity marker correlating with local stiffness and elastin preservation, as seen in BAV‐A patients. This complements work by Stern et al. (2019) which demonstrated a strong osteopontin (OPN) signal and calcification‐linked remodelling, whereas TAV‐A had medial degeneration with collagen and elastin degradation. Similarly with mechanical properties, age appears to be more important that BAV vs. TAV phenotype for the ultimate strength of aortic tissues (García‐Herrera et al., 2012), and BAV‐A tissue was found to be stiffer relative to TAV‐A tissue with a similar aortic diameter (Chim et al., 2020).

These differences in tissue structure between BAV‐ and TAV‐associated aortopathy likely arise through complex interactions between haemodynamic forces and underlying biological susceptibility rather than either factor acting in isolation. In BAV, eccentric systolic flow and altered wall shear stress produce region‐specific endothelial activation that influences vascular smooth muscle cell phenotype, extracellular matrix turnover and mechanosensitive signalling pathways. These haemodynamic stimuli interact with intrinsic developmental and genetic factors, including dysregulated transforming growth factor β (TGF‐β) signalling, to drive regional remodelling of the aortic wall. Conversely, in TAV‐associated disease, physiological ageing, cardiovascular risk factors and chronic alterations in vascular homeostasis appear to predominate, promoting endothelial dysfunction, matrix metalloproteinase activation and progressive extracellular matrix degeneration. Oxidative stress has also emerged as an important contributor to thoracic aortic aneurysm pathobiology. Oxidative stress reflects an imbalance resulting in increased levels of reactive oxygen species via over‐production, and/or impaired antioxidant activity. Oxidative stress within the aortic wall causes altered inflammatory signalling including macrophage infiltration and matrix metalloproteinase (MMP) activation, resulting in changes within the aortic ECM including elastin and vascular smooth muscle cell (VSMC) degradation (Kazaleh et al., 2023). A greater impairment in antioxidant response, identified by an increase in superoxide production (Billaud et al., 2017) and reduced expression of endothelial nitric oxide synthase (Aicher et al., 2007) has been identified in BAV aortic tissue compared with TAV. As the primary focus of this review is on the structural and biomechanical characterisation of aortic tissue, readers are referred to dedicated reviews for a comprehensive discussion of oxidative stress and redox signalling in thoracic aortic disease (Kazaleh et al., 2023; Portelli et al., 2021).

Although these molecular pathways converge on common processes including elastin fragmentation, collagen remodelling and smooth muscle cell dysfunction, the relative contribution of haemodynamic and intrinsic biological mechanisms is likely to differ between BAV and TAV aortopathy, providing a potential explanation for their distinct structural and biomechanical phenotypes.

There are some limitations that should be noted in the interpretation of the available evidence in Table 1 due to recurring methodological limitations. Most studies are retrospective and rely on surgically resected tissue, and hence there is selection bias towards patients with advanced disease requiring surgical intervention. Histological analyses are frequently performed on only a subset of the population cohorts noted in the papers because of tissue availability, quality, or incomplete pathology records. As noted in Table 1, some studies also contain inconsistencies in reporting, including discrepancies between patient numbers described in the text, tables and figure legends, making accurate data extraction challenging. In addition, most cohorts are heavily male dominated, reflecting the epidemiology of aortic aneurysms but limiting the generalisability of findings to female patients. Comparisons between BAV and TAV are further complicated by the consistently younger age of BAV patients, which may confound differences in extracellular matrix composition, elastin integrity, collagen remodelling and other age‐dependent histopathological features. Finally, heterogeneity in tissue sampling location, histological grading systems, staining protocols and quantitative methodologies limits direct comparison between studies.

## 2 MATURATION DEFECT VERSUS AGE‐RELATED DEGENERATION

Grewal et al. (2014) examined wall biopsies from the ascending aorta of BAV patients with and without dilatation, and TAV patients with and without dilatation. Through examination of VSMC differentiation (e.g., α‐smooth muscle actin, calponin), lamin A/C (a protein important for VSMC maturation) and progerin (a protein linked to cellular ageing), the authors explored whether BAV was associated with structural immaturity compared to TAV patients. The TAV aneurysmal tissue samples were found to have greater signs of the normal ageing process (higher progerin and inflammation). In contrast, the BAV tissue had a thinner intima and a lower expression of smooth muscle differentiation markers and lamin A/C. The authors concluded that BAV‐A occurs due to an intrinsic maturation defect of the aortic wall, not only because of secondary haemodynamic stress. This intrinsic ‘structural immaturity’ was suggested to be a likely contributor to the greater propensity for aortic dilatation and dissection seen in BAV patients. Hence, this highlights the need for differential diagnostic and therapeutic strategies for BAV patients relative to TAV‐associated aortic disease. The authors later conducted a unique lifespan histological analysis of the ascending aorta in TAV and BAV populations, examining non‐dilated specimens from fetal life to adulthood (Grewal et al., 2020). In TAV, a structured three‐phase trajectory was identified: postnatal maturation characterised by increasing elastic lamellae and medial thickening, a stabilisation phase during childhood and adolescence, and eventual adult degeneration marked by lamellar loss and intimal expansion. In contrast, BAV demonstrated a fundamentally different developmental course. They found prenatal intimal thickening in BAV, followed by postnatal thinning and an absence of the orderly maturation–stabilisation sequence observed in TAV. Elastic lamellar accrual occurred later and plateaued without the subsequent age‐related decline seen in TAV. Importantly, BAV did not exhibit the progressive degenerative changes characteristic of physiological vascular ageing in TAV. These findings (represented schematically in Figure 3) support the concept that BAV‐A arises from an intrinsic disturbance of vascular maturation rather than an acceleration of normal ageing processes, providing a developmental framework that complements biochemical evidence of smooth muscle cell immaturity and reduced lamin A/C expression described in their earlier work (Grewal et al., 2014).

**FIGURE 3:**
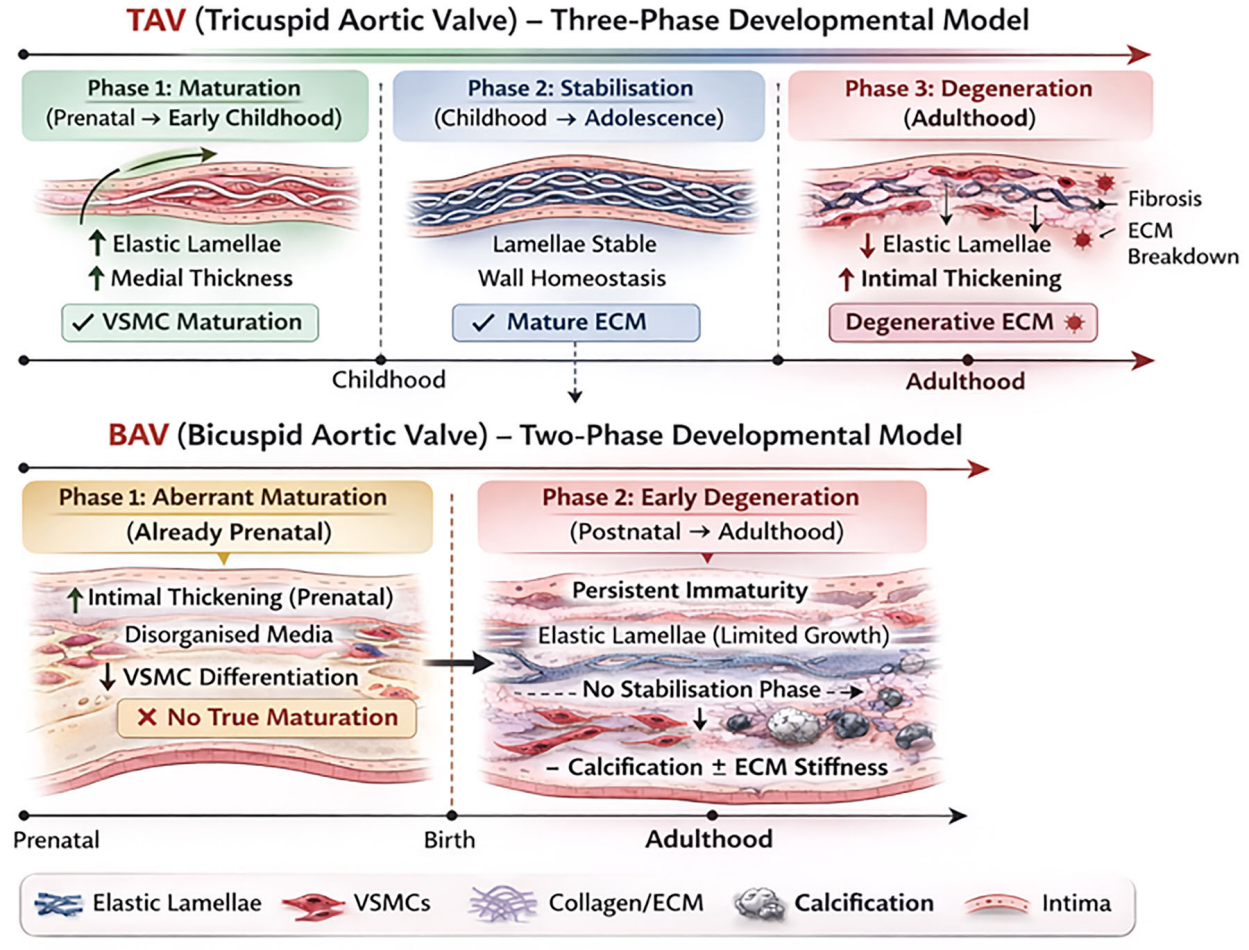
Schematic representation of the developmental trajectories proposed by Grewal et al. (2020) for tricuspid (TAV) and bicuspid (BAV) aortic wall. For the TAV panel, Phases 1 and 2 are supported primarily by the lifespan histological study of Grewal et al. (2020), while Phase 3 is supported by evidence of medial degeneration, ECM remodelling and biomechanical deterioration from Collins et al. (2008), Phillippi et al. (2014), Grewal et al. (2014), Stern et al. (2019), Karalko et al. (2021), Mennander et al. (2022), Tucker et al. (2022) and Zhang et al. (2024). For the BAV panel, Phase 1 is supported by Grewal et al. (2014, 2020), while Phase 2 is supported by Chim et al. (2020), Phillippi et al. (2014), Grewal et al. (2020), Stern et al. (2019), Fletcher et al. (2022), Tucker et al. (2022) and Zhang et al. (2024).

In summary, we can argue that across the last two decades, ageing has emerged as a stronger determinant of aortic wall degeneration and mechanical weakness in TAV‐A as compared to BAV‐A. Multiple histopathological studies show that medial degeneration severity, fibrosis, smooth‐muscle cell loss and inflammatory features increase with age in TAV patients, whereas these relationships are weak, inconsistent or absent in BAV cohorts. These trends are reinforced by biomechanical data which show age‐dependent reductions in delamination resistance and tissue integrity in TAV‐A as compared to BAV‐A tissue at comparable aortic diameters (Figure 4). Collectively, these findings support a model in which TAV‐A follows a progressive, age‐linked degenerative trajectory, whereas BAV‐A frequently dilates in the relative absence of classical age‐driven medial failure, instead reflecting phenotype‐specific remodelling processes that are not simply accelerated ageing.

**FIGURE 4:**
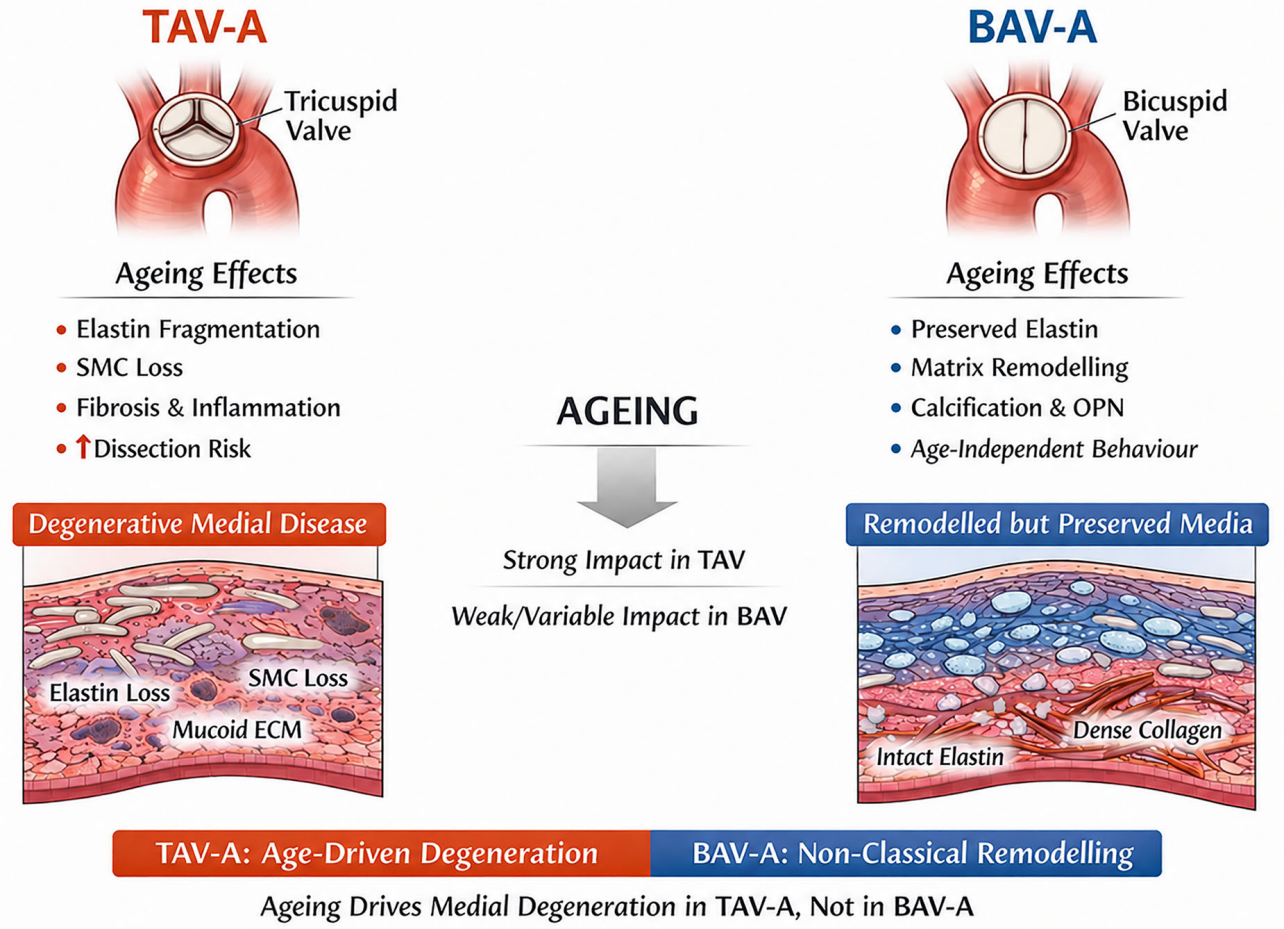
Idiopathic degenerative TAV‐A is driven by age‐related medial degeneration as compared to BAV‐A, which exhibits remodelling by preserved media.

## 3 GENETICS AND ATAA

Whilst this review focusses largely on histopathology, biochemical and biomechanical differences in BAV, and idiopathic degenerative ATAAs, it is important to highlight the importance of considering genetics. Strong hereditary influence is well‐established in ATAAs, particularly in syndromic and familial forms involving extracellular matrix proteins, TGF‐β signalling and smooth muscle contractile genes (Isselbacher et al., 2016). BAV‐A sits within this genetically influenced spectrum but differs fundamentally from TAV‐A. BAV demonstrates specific, albeit heterogeneous, genetic associations including variants in NOTCH1, SMAD6 and GATA family genes implicating abnormal outflow tract development and vascular maturation (Bravo‐Jaimes & Prakash, 2020). In contrast, most TAV‐A, outside recognised syndromes lack clear Mendelian drivers and are more strongly linked to ageing, hypertension and acquired medial degeneration (Isselbacher et al., 2016). These divergent genetic architectures further suggest that BAV aortopathy reflects a developmental aortopathy with intrinsic smooth muscle and extracellular matrix immaturity, whereas TAV aortopathy more commonly represents a degenerative process. Consequently, differences in genetic substrate may underpin distinct natural histories, biomechanical trajectories and responses to haemodynamic stress, with implications for surveillance thresholds and risk stratification.

## 4 LIMITATIONS OF THE EXISTING STUDIES

Most of the studies utilise aortic tissue harvested at the time of surgery, which is inherently biased towards advanced disease and hence does not capture any early potential mechanobiological biomarkers. Biomechanical testing in vitro is quite varied with different techniques utilised and often studies being limited to small sample cohorts, which is often due to difficulties in obtaining tissue during elective surgery. Histological grading systems utilised in many studies are semi‐quantitative and composite, aggregating features with distinct mechanical relevance and limited spatial resolution, which complicates direct translation to potential biomechanical risk. In addition, in many studies biochemical and histological analyses are rarely integrated directly with mechanics in the same specimens, meaning that causal links are inferred. Finally, there are significant differences in how and from where aortic samples are taken (e.g., root vs. mid‐ascending), and hence this may further limit comparability across studies and help to explain any conflicting trends in the literature. Figure 1 highlights the different clusters of BAV‐A and valve morphologies. However, a key limitation of the current literature is the limited stratification of BAV aortopathy according to valve morphology. Although clinical studies suggest that specific valve conditions are associated with more malignant outcomes, relatively few biomechanical or biochemical studies investigate these subgroups separately. The precise tissue sampling and information of valve morphology are not always provided or available in the studies covered in this review.

In terms of this review, a comprehensive overview of all biomechanical papers which have focussed on BAV‐A are not provided; rather it highlights those where BAV‐A and TAV‐A biomechanical properties have been characterised alongside histological or biochemical properties. Detailed examination of valve and aortopathy phenotypes as compared to histopathology and biomechanical properties and genetics is beyond the scope of this review.

## 5 CLINICAL OUTLOOK AND FUTURE DIRECTION

Collectively, these basic science studies support a shift away from aortic aneurysms being seen as a homogeneous disease group but rather towards phenotype‐informed risk stratification. The evidence suggests that with idiopathic TAV‐A the focus should be on age‐related degeneration and dissection vulnerability, whereas for BAV‐A studies should focus on better understanding biological remodelling and its functional manifestations. Importantly, multiple independent studies show that aortic diameter correlates poorly with histological severity or biomechanical vulnerability, particularly in BAV, reinforcing the limitations of diameter‐only surgical thresholds. Future progress will depend on prospective, longitudinal studies integrating in vivo imaging, *ex vivo* mechanics and biochemical/molecular profiling from the same patients, enabling clinical translation to move away from descriptive pathology to predictive mechanobiology. Of note, for example, is advancement in molecular imaging such as fluorine ^18^F‐labelled sodium fluoride positron emission tomography (PET), which can identify a diseased aortic wall in BAV patients independent of aortic diameter (Nash et al., 2026). This is an important development because it provides an in vivo biomarker of wall biology which is associated with the tissue level measurements (Fletcher et al., 2022) highlighted in this review, namely medial elastin fragmentation, microcalcification and stiffness. Whilst blood‐based biomarkers have largely been found to be inadequate for aortic aneurysm surveillance, recent advances with plasma desmosine, which is a highly specific marker of elastin degradation, are particularly exciting. These advances are reviewed comprehensively by Gombert et al. (2026). In the surgical setting, point of care devices which can provide real‐time information about tissue structural integrity may guide surgical intraoperative decision‐making (Davies et al., 2021). Future basic science work on BAV should focus on further unravelling the link between genetics, valve morphology and the principal patterns of BAV aortopathy.

## AUTHOR CONTRIBUTIONS

All authors should have been involved in the writing of the paper. All authors have read and approved the final version of this manuscript and agree to be accountable for all aspects of the work in ensuring that questions related to the accuracy or integrity of any part of the work are appropriately investigated and resolved. All persons designated as authors qualify for authorship, and all those who qualify for authorship are listed.

## CONFLICT OF INTEREST

None declared.

## GENERATIVE AI STATEMENT

During the preparation of this work, the authors used ChatGPT 5.2 (OpenAI) to generate Figures 1, 2, 3, 4 and to summarise key findings from the papers reviewed in Table 1. After using this tool, the authors reviewed and edited the content as needed and take full responsibility for the content of the publication.
